# Do political parties always prefer loyalists? Evidence from South Korea

**DOI:** 10.1371/journal.pone.0291336

**Published:** 2023-11-02

**Authors:** Myeonghwa Lee, Shang E. Ha, Wonjae Lee

**Affiliations:** 1 Graduate School of Cultural Technology, Korea Advanced Institute of Science and Technology, Daejeon, Republic of Korea; 2 Department of Political Science, Sogang University, Seoul, Republic of Korea; University of City Island, CYPRUS

## Abstract

This paper examines the relationship between ideological polarization and party disloyalty, focusing on the moderating role of the status of a political party in the legislature, i.e., the ruling party or the opposition party. It hypothesizes that the ruling party is willing to endorse disloyal candidates whose issue positions are not close to their own party’s platform, whereas the opposition party is likely to punish disloyal candidates to demonstrate party unity in the nomination process. The present study tests this hypothesis, using data from South Korea, where the nomination process for the parliamentary election is dominated by party leaders. The results are by and large consistent with the hypotheses. In line with previous studies, our results suggest that party loyalty is one of the driving forces of polarization in politics. In order to fully understand party polarization at the level of political elites, it is necessary to consider heterogeneous effects of party members’ behavior on candidate selection, varying across the party’s status, either the ruling party or the opposition party.

## Introduction

The paper addresses the issue of increasing ideological polarization among representatives in legislative assemblies in many democratic countries. Despite the lack of consensus regarding its causes, it is widely acknowledged that ideological polarization can have severe negative consequences, such as the rise of populism, which can make democratic institutions vulnerable and even prone to collapse [1, 2]. Consequently, the existing literature on democratic erosion calls for solutions to mitigate ideological and affective polarization among lawmakers and ordinary citizens in order to uphold democratic values [1, 3, 4].

Theoretical research suggests that polarization in the legislative branch may be reinforced because lawmakers seeking re-election typically need to align with their own party’s policy positions [3, 5, 6]. This is especially true when the nomination process is controlled by party leaders, who have the power to deny nominations to incumbents whose issue positions diverge from the party’s platform. Once polarization has begun, it tends to be reinforced as parties are inclined to penalize disloyal candidates and reward loyal ones in the subsequent election [7].

Although the argument for the role of party loyalty in polarization seems compelling, it is important to consider the possibility of heterogeneous behaviors by political parties, which may vary depending on their status in the political arena. For instance, a ruling party may be more tolerant of dissenting opinions from incumbents or potential candidates, unless such opinions alienate the core supporters. Moreover, appealing to ideologically moderate voters is crucial for determining the electoral outcome, even in a polarized era. Hence, a ruling party may embrace candidates whose positions are not necessarily consistent with those of the party to expand its range of supporters. Conversely, the opposition party may require its candidates to adhere strictly to the party platform, as building a unified voting bloc is important for winning the election. Being open to ideological diversity may not be a good strategy for the opposition party, as it may confuse the voters.

In summary, this paper investigates the relationship between ideological polarization and party disloyalty, with a focus on the moderating effect of a political party’s status in the legislature. The hypothesis is that the ruling party is more likely to tolerate disloyal candidates, while the opposition party is more likely to punish them. The study uses data from South Korea, where the nomination process for the parliamentary election is dominated by party leaders. To measure the degree of loyalty of lawmakers, the paper uses a new method called SDSM Backbone Networks [8, 9]. The findings of this study are expected to contribute both substantively and methodologically to the existing literature on ideological polarization among political elites.

## Theory and hypothesis

Ideological polarization in the legislative branch is a well-documented phenomenon in political science. This trend is particularly evident in the United States, where Republican congresspersons have shifted towards conservatism and Democratic congresspersons towards liberalism over time [3, 10–12]. While scholars and commentators agree that ideological polarization in the US Congress emerged in the late 1970s or early 1980s, the root causes of this polarization are still being investigated. Polarization in Congress can be measured by analyzing the roll-call votes of each lawmaker over time [13–15]. Approximately four decades ago, the most conservative Democratic congressperson was more ideologically conservative than the most liberal Republican [9, 12, 14, 16, 17]. However, today, the most conservative Democratic congressperson is more ideologically liberal than the most liberal Republican. This polarization has reduced the possibility of compromise and led to frequent deadlocks. Similar trends of polarization in the legislative branch have also been reported in other democratic countries [18, 19], and it is believed to contribute to ideological and emotional polarization among voters.

The measurement of ideological polarization among political elites, such as representatives, is commonly done through analyzing their roll-call votes in the legislative branch. This is because the content of a bill is often ideological, and whether a lawmaker supports or opposes it can be categorized as conservative or liberal. By examining a complete set of roll-call votes, a lawmaker can be placed on a one-dimensional (or two-dimensional, economic dimension versus social dimension) ideological spectrum. Alternatively, co-sponsorship patterns can also be used to measure polarization [20, 21]. In the US Congress, bills are often sponsored by both Democratic and Republican lawmakers (known as "bipartisan bills"), but some bills are proposed by only one party. If the number of Democrats (or Republicans) who sponsor bills proposed by Republicans (or Democrats) decreases over time, it is indicative of polarization, as cooperation and coordination between the two major political parties become increasingly rare.

The trend of party-line voting in the legislative branch has drawn the attention of pundits and commentators [9, 17, 22, 23], who see it as a symptom of polarization. As a party’s platform becomes more ideologically extreme, lawmakers may have little incentive to deviate from it. However, despite the apparent intensification of party-line voting, some lawmakers still hold positions that do not align with their party’s platform. Lawmakers have two goals: getting reelected and promoting their status in the party. If reelection is unlikely or promotion is impossible, they may behave independently of their party [24–26]. In such cases, political parties have little recourse because these lawmakers will soon retire. However, competent lawmakers who are likely to be reelected and promoted may challenge party leadership.

Why do political parties tolerate disloyal members? In political systems with weak parties, such as the United States [27], lawmakers, particularly in the House of Representatives, prioritize the interests of their constituents over the national interest. Therefore, they may deviate from the party platform if it does not align with the preferences of their district’s voters [28, 29]. In weak party systems, party leaders do not have the power to effectively discipline disloyal members because they do not dominate the nomination process. If disloyal members, who are popular in their districts, continue to win elections, they may still contribute to the party and be valued by party leadership.

The decision of party leaders to keep disloyal members in the party varies depending on the status of the party in the political arena [30–33]. In the case of a ruling party, dissenting opinions from its members may be tolerated unless they seriously damage the party’s reputation. Disloyal lawmakers or candidates who represent extreme positions in their districts can be seen as an asset by the ruling party, as they may attract additional supporters among the electorate [12, 34, 35]

On the other hand, the opposition party tends to prioritize party unity. Clarity in terms of ideology and issue positions is necessary to build a unified voting bloc, which can lead to a successful election outcome. In some cases, however, disloyal members may be kept in the opposition party if they represent a key demographic or a swing district, and their departure from the party could potentially hurt its chances of winning.

Based on the theoretical considerations above, we offer the following hypotheses:

**H1. Disloyal members of the ruling party are more likely to be rewarded than their counterparts in the nomination process.**
**H2. Loyal members of the opposition are more likely to be rewarded than their counterparts in the nomination process.**

We tested these hypotheses using data from the National Assembly of South Korea. In contrast to the US Congress, the South Korean legislative branch does not use roll-call votes for all bills under consideration. Instead, members of the National Assembly can vote for or against bills dealing with sensitive or controversial issues without disclosing their names. Therefore, we relied on an alternative method to measure levels of polarization in the legislature, utilizing a bill co-sponsorship network to indicate how closely each lawmaker’s issue position aligns with the party’s majority opinion.

## Measuring loyalty using SDSM backbone networks

This section introduces a novel approach for measuring individual loyalty within a political party, based on the analysis of inter-member relationships. Prior to presenting our proposed method, we conduct a comprehensive examination of the current discourse surrounding loyalty and its established measurement techniques. Our approach effectively captures the salient characteristics of loyalty that foster party cohesion and solidarity.

Party loyalty is the act of maintaining or strengthening the party unit and making campaign contributions [18, 35–37]. On this basis, party elites and voters decide whether to reward or punish individual politicians. As loyalty is significantly related to the careers of lawmakers, it is possible to understand and predict the behavior of incumbent lawmakers by investigating loyalty and its consequences. Party loyalty has been measured using roll-call votes in the legislative process in terms of whether lawmakers follow the party line using a dichotomous value [18] or by counting the instances of voting for or against [30, 32, 33]. In addition, financial contributions also reflect loyalty [36].

We refine existing measures of party loyalty by considering intra-party and inter-party relationships separately. Even if a lawmaker agrees with most of the party’s views, this may not be evidence of loyalty if he or she votes for the same number of bills proposed by other party members. To address this issue, we make co-sponsorship of bills conditional on party affiliation. In doing so, we suggest that party loyalty is twofold: intra-party preference and inter-party avoidance.

**Fig 1:**
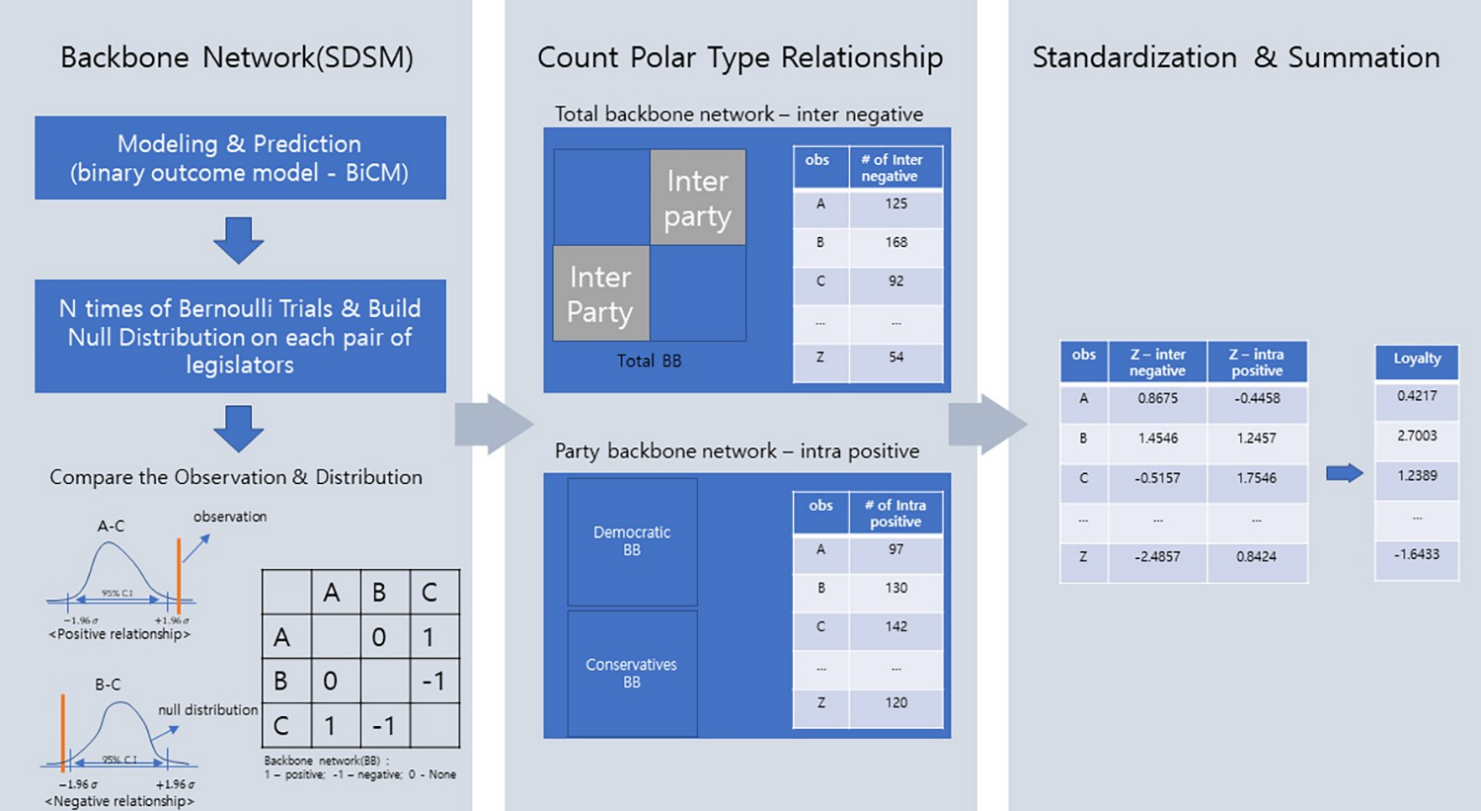
Process of obtaining the loyalty score of individual lawmakers. It has three major steps quantifying party loyalty we suggest, which is (1) extracting backbone of legislative network, (2) counting inter and intra relationship which is the result of previous step and lastly, (3) standardizing counting values and finalize the scoring by adding each variable.

Relationship-based party loyalty is processed from the bipartite network of bill sponsorship, where lawmakers and bills are the nodes (Fig 1). Edges exist when lawmakers sponsor a bill without weight. To obtain the relationship between the representatives, we extract a backbone network (BB) whose edges represent the relationship. There are three different signs in the network: positive (+1), moderate (0) and negative (-1). We apply the Stochastic Degree Sequence Model (SDSM) [9] to obtain the BB. If we simply count the number of co-sponsored bills between two politicians to determine the relationship, this can lead to distortions in the interpretation of weighted edges, as higher degree agents (lawmakers) will necessarily have stronger edges than lower degree agents. As the number of bills proposed varies from person to person, this should be standardized. Researchers have found a number of backbone relations that can solve this problem [38, 39]. Among these approaches, SDSM conditions both the degrees of agents and artefacts (legislation), thus addressing the overconditioning problems and computational complexity [8]. The observed bipartite network is considered as one of many possible outcomes of an unobserved stochastic process of agent-artifact matching. Using a statistical model to predict whether lawmakers will sign a given bill, the stochastic co-sponsorship distribution of the two representatives is determined. If the observed value lies outside the confidence interval of the conditional null distribution, a relationship is assumed to exist [8].

To obtain the measure of party loyalty, the following three main steps are taken. First, we construct two different BBs using SDSM. One is a total backbone network (total BB), where all members of the National Assembly and legislature are included for the nodes of the input bipartite network and comparative relationships between politicians are the output. The other is a party backbone network (party BB), where specific party members and their sponsored bills are the input.

Second, we count the number of positive edges in Party BB for each person, i.e. the number of intra-positive relationships. For the inter-negative relationship, we count the number of negative edges in the total BB when two lawmakers have different party affiliations. It is possible to obtain individual intra-positive relationships from the total BB without splitting the data. In this case, however, there is little variation in the number of intra-positive edges from the same party due to the large number of intra-party collaborations.

Finally, we standardize the two extracted values. We transform the two variables to follow a standard normal distribution with zero mean and unit variance and score them by summing the values. The scaling of the values takes into account the difference between the distributions of the number of intra-positive and inter-negative relationships. This is used to calculate the degree of party loyalty of lawmakers, taking into account the polar nature by assessing how and with whom individuals maintain relationships. It also reveals intrinsic connections through cumulative behavior.

Following and adapting a previous study [9] that revealed the increase in polarization over time, we propose a new method for calculating party loyalty. In doing so, we examine how Korean lawmakers’ decisions have been rewarded/sanctioned over the course of the last two legislative periods.

This study will become one of the various approaches to interpreting the outcomes of party loyalty observed not only in the United States but also in different countries. It can be interpreted through dimensions of the party as an entity or on an individual level, among other diverse perspectives [2, 40]. To test our hypotheses, we use co-sponsorship data from the 19th and 20th South Korean National Assembly. In the 19th term (June 2012-May 2016), the conservative Saenuri Party (New Frontier Party) was the ruling party and the Democratic Party was the opposition. In the 20th term (June 2016—May 2020), the Democratic Party was the ruling party and the Mirae Tonghap Party (United Future Party; formerly the Saenuri Party) was the opposition. During these periods, the two ideologically contrasting parties (conservative and democratic) alternated the roles of ruling party and main opposition party with a marginal difference in seats. In order to capture the effects of intra- and inter-party relationships, we include only two major parties in our analysis.

**Fig 2:**
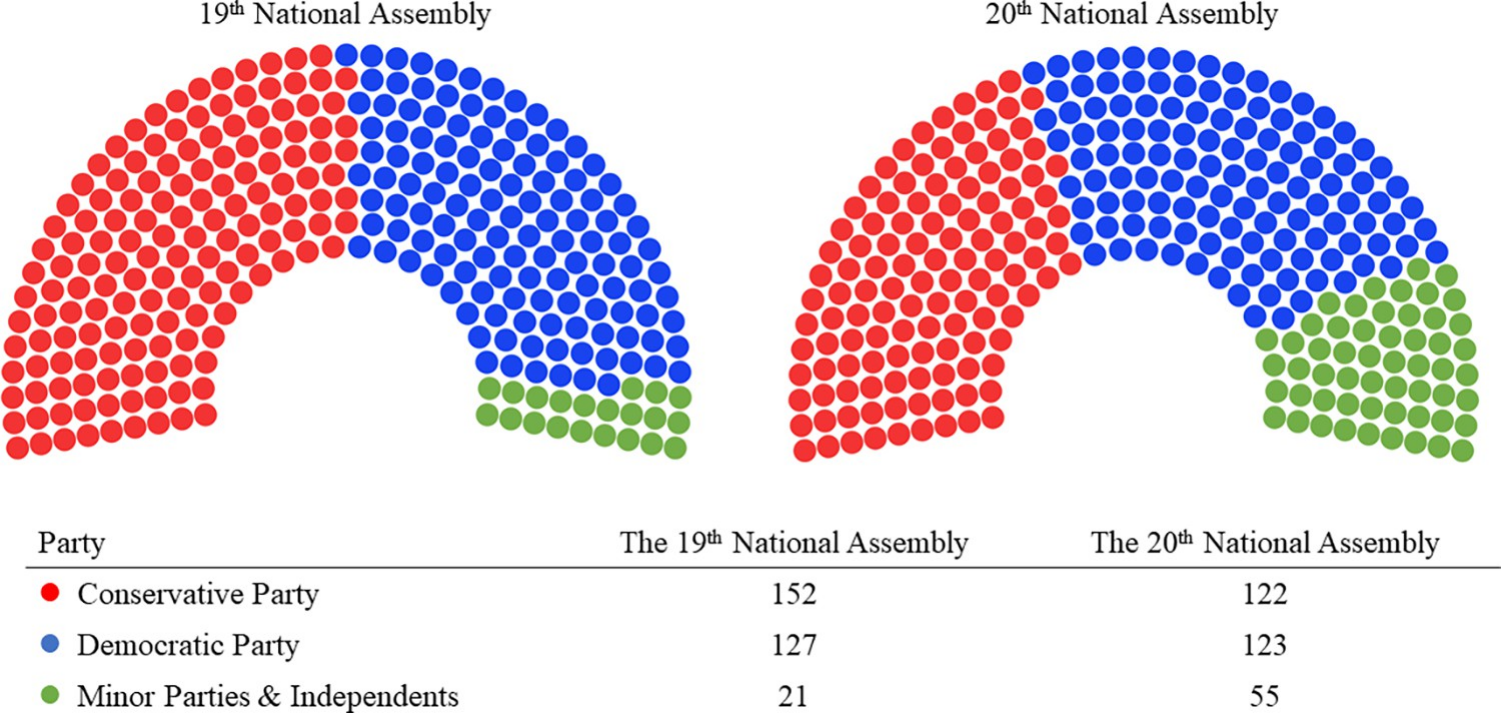
Visualize the number of seats in each 19th and 20th National Assembly in Korea. The figure was created using a visualization tool on the site (https://app.flourish.studio/). It represents the seat distribution of the 19th and 20th political parties in South Korea, where red represents conservative parties, blue signifies progressive parties, and individuals not affiliated with either major party are depicted in a different color.

The two dominant political party immediately follows as the result of the general elections for the 19th and 20th terms of the Korean National Assembly (Fig 2). Non-affiliated independent candidates and members of smaller parties are aggregated into a single group. Notably, the 20th term began with the Conservative Party in power due to the presidency of Geun Hye Park. However, following her impeachment, the Democratic Party gained the majority and assumed control in 2017.

Throughout history, the two dominant political parties have engaged in perpetual conflict, relegating minor parties to the status of mere satellites rather than independent entities. Consequently, we exclusively consider the two major parties as the primary drivers of polarization.

To accurately evaluate an individual lawmaker’s loyalty in relation to their nomination for the next election, it is essential to consider that loyalty can fluctuate over the course of their term. To this end, we divide the term into four years, and construct four bipartite networks (BBs) by aggregating the lawmaker’s legislative activity up to that point in time. The number of lawmakers exhibiting signal relationships (positive or negative) increases over time within the overall BB. Notably, the number of polarized relationships based on the cumulative co-sponsorship data for each National Assembly increased by 30.96% (from 43,609 to 57,114) and 24.14% (from 41,120 to 51,048) compared to the number of relationships identified from the one-year bill network. In the 19th National Assembly, the BB with the lowest number of positive relationships between members of different parties was derived from the bill sponsoring data up to the second year, while in the 20th National Assembly, this number was smallest in the first-year bill sponsoring data. These observations contrast with the overall trend of increasing polarization within each National Assembly over time. Furthermore, our analysis reveals the presence of atypical members with remarkably low levels of party loyalty.

### Data

We collected data on lawmakers’ bill sponsorship from the official Korean online bill information site (https://likms.assembly.go.kr/bill/main.do) covering the 19th and 20th National Assembly legislations proposed from June 2012 to May 2020. In addition, we obtained the demographic information of representatives from the National Election Commission site (http://info.nec.go.kr/), which serves as the official source of candidate information. To ensure the validity of our analysis, we excluded political actors who were not affiliated with the two major parties (Conservative Party and Democratic Party) at the time of the general election and just before the candidate nomination announcement. Furthermore, we did not include bills proposed after the nomination announcement, as these occur prior to the final day of the Assembly term and the short time interval does not significantly impact party loyalty. Additionally, issues such as party defections following the nomination announcement may result in missing data, which could potentially skew the phenomenon we seek to verify.

### Measures

We investigated how individual party loyalty influences the probability of nomination in future general elections by party leaders in Korea. Nomination decisions can be classified into three categories: cut-off, competition-nomination, and single-nomination. Cut-off deprives lawmakers of the opportunity to participate in the election under their party’s banner. If a lawmaker is cut-off, he or she must give up running for reelection as a party member. This is the most severe decision that party leadership can make against an incumbent Assembly member. Competition-nomination allows lawmakers to participate in primary elections to determine the final electoral candidate. This is not necessarily viewed as a reward, as the intention of the party leadership can be either compensatory or punitive, depending on the subject and situation. Lastly, single-nomination occurs when only one candidate desires to run for a district and party leaders accept them as their party’s representative. This is viewed as the most favorable reward because party leaders may have candidates run in primary elections or cut them off if they are not satisfied with a volunteer candidate. Among these three categories, we chose cut-off and single-nomination as our dependent variables to identify the relationship between party loyalty and compensation or sanction in electoral success.

We developed two distinct models with identical independent and control variables, but with different binary dependent variables. The first model predicts single-nomination, while the second model predicts cut-off. Our main independent variable is relationship-based party loyalty, which we measured for members of the two major parties in the incumbent Assembly. We processed this variable using the bill-sponsorship and politicians’ backbone network, and expressed it as a numerical value following a normal distribution with a mean of zero. We also accounted for fluctuations in party loyalty during the Assembly’s term by extracting the values separately for each year.

Control variables related to individual lawmakers and their nominations were also included in our analysis. We considered six covariates, which are as follows: low participants, local, male, super senior, 20th term, and betweenness centrality. We provide a brief description of each variable below.

"*Low participation"* is an indicator that measures the level of involvement of lawmakers in the legislative process. Specifically, it takes the value of 1 if the ratio of the bill sponsorships that a lawmaker participated in is less than the bottom 5% of the distribution among party members, and 0 otherwise.

"*Proportional*" is an indicator that distinguishes between proportional representatives and constituency representatives. A value of 1 is assigned if the lawmaker was elected as a proportional representative, and 0 otherwise.

"*Gender*" is an indicator variable that reflects the lawmaker’s sex: 1 for male and 0 for female.

"*Super Senior*" is an indicator that identifies members of the National Assembly who have been re-elected for five or more terms. It takes the value of 1 for such members and 0 otherwise.

"*20th Term*" is an indicator that takes the value of 1 for lawmakers in the 20th National Assembly and 0 for lawmakers in the 19th National Assembly.

"*Betweenness*" refers to the measure of a lawmaker’s betweenness centrality in the bill-sponsoring network, quantified by their ability to serve as a bridge between different groups of lawmakers. We calculated the betweenness centrality by projecting the original bipartite network into a one-mode network.

In addition to party loyalty, it is worthwhile to consider the measure of betweenness centrality in our model. Many researchers have viewed betweenness as an indicator of power within the Assembly [41–45]. Additionally, politicians with high betweenness centrality often serve as bridges between polarized parties. However, betweenness centrality alone may not fully account for an individual actor’s boundary-spanning ability in cases of fractional conflict within a party. The National Assembly of Korea has experienced significant discord within political parties, and the betweenness within a party is as significant as between parties. Therefore, it is necessary to control for betweenness if we wish to examine the effects of party loyalty independently.

We did not take into account the party affiliation of the National Assembly members, as the term in which they served represents the political ideology in the dataset. For example, during the 20th National Assembly, the ruling party was the Conservative party, while the opposition was the Democratic party, whereas during the 19th National Assembly, the Democratic party was the ruling party. We also track the proposed date of each bill and divide the dataset into chronological order. We set four target dates on a yearly basis from the date of the National Assembly’s inauguration. Each dataset has a network of bills and lawmakers before each target date. Loyalty and betweenness centrality are calculated separately based on the target date, resulting in four different values for each variable.

Tables 1 and 2 present the descriptive statistics and correlations of the two datasets. We extracted their loyalty and betweenness centrality based on the bill sponsorship information up to the second year after the inauguration of the 19th and 20th National Assembly.

**Table 1:** Descriptive statistics of ruling party data.

	mean	sd	min	max	1	2	3	4	5	6	7
**single**	0.3887	0.4884	0	1							
**loyalty**	0	1.4349	-8.9175	3.1022	-0.0358						
**Low Participants**	0.0526	0.2238	0	1	-0.0763	-0.5513					
**Proportional**	0.8623	0.3452	0	1	0.2221	-0.0456	-0.0111				
**Gender**	0.8543	0.3536	0	1	0.1175	-0.0128	-0.0054	0.4011			
**Super senior**	0.0445	0.2067	0	1	-0.1319	-0.4593	0.3886	0.0863	0.0336		
**20th Term**	0.4575	0.4992	0	1	0.2848	0	0.0019	0.1074	-0.1043	0.0381	
**Betweenness**	6.2908	6.6859	0.0216	56.2991	0.0657	0.0631	-0.1956	-0.0068	-0.0065	-0.1165	0.1185

It describes the basic descriptive statistics of variables in the ruling party dataset and its correlation on each pair of variables. It shows that there is no outstanding linear relationship between any pair of variables.

**Table 2:** Descriptive statistics of opposition party data.

	mean	sd	min	max	1	2	3	4	5	6	7
**cutoff**	0.1538	0.3617	0	1							
**loyalty**	0	1.3514	-6.3284	2.6773	-0.0634						
**Low Participants**	0.0529	0.2243	0	1	-0.0412	-0.3908					
**Proportional**	0.8269	0.3792	0	1	-0.0515	-0.2393	0.0513				
**Gender**	0.8221	0.3833	0	1	-0.0107	-0.2233	0.0537	0.4186			
**Super senior**	0.0529	0.2243	0	1	0.1374	-0.0687	0.1361	0.1081	0.0537		
**20th Term**	0.5192	0.5008	0	1	0.0636	0	0.0124	0.043	0.106	0.0124	
**Betweenness**	5.5566	5.1588	0.0952	36.295	-0.1125	-0.0956	-0.1534	-0.0091	0.0661	-0.0671	0.1062

It describes the basic descriptive statistics of variables in the opposition party dataset and its correlation on each pair of variables. It shows that there is no outstanding linear relationship between any pair of variables.

### Model

We present our statistical models examining relationship-based party loyalty and public nomination for the next National Assembly. We utilized two logistic regression models with different binary dependent variables—a rewarding model for ’single-nomination’ and a sanction model for ’cut-off’. In cases where there was conflict in the nomination decision during the review process, we only included the final announcement in our analysis.

We divided the input data into two datasets based on the party affiliation of each lawmaker from the 19th and 20th National Assembly. The governing party dataset includes Conservative party members from the 19th National Assembly and Democratic party members from the 20th National Assembly, while the opposition party dataset includes all other members. We applied each dataset to the respective model, with lawmakers who served for both terms included twice and those who served for only one term included only for the term they served.

We are interested in investigating whether party loyalty is rewarded or punished within political parties. To test our hypotheses, we have taken two main approaches. First, we expected that disloyal lawmakers would be rewarded with a ’single nomination’ in the next general election if they are part of the governing party. However, if they belong to the opposition, we anticipated that they would be punished by being deprived of the opportunity to be named on ballots due to their lack of loyalty to the party.

In contrast to other methods of measuring party loyalty such as roll call voting or floor voting, we utilized a collective loyalty approach that considers relationships between and within party groups. Through this approach, we were able to give lawmakers a score based on their ability to maintain friendly relationships with their party colleagues.

For each specified model, we additionally included the interaction between loyalty score and the low participants variable. As the number of participations in the legislation is used in the process of constructing a loyalty score, the lawmakers with an extremely small participation rate are expected to apply stricter relationship decisions than other lawmakers.

We further illustrate the instances in which compensation and punishment are determined at the party level. To accomplish this, we employ the same models as described previously, but utilize the party loyalty score based on a yearly unit. These models are categorized by year since party loyalty may differ from the overall relationship before the nomination announcement. By adopting this approach, we can shed light on the discrepancies in results between the government and opposition party, and the timing of the decision-making process.

## Results

Table 3 presents significant outcomes from each model-data combination.

**Table 3:** Logistic regressions predicting candidate nominations of the two major parties in the Korean 19th and 20th National Assembly.

	Dependent variable:			
	Single Nomination in Ruling Party		Cutoff in Opposition Party	
	(1)	(2)	(3)	(4)
loyalty	-0.392*	-0.402**	-0.348^+^	-0.386*
	(0.153)	(0.156)	(0.179)	(0.195)
Low Participation	-2.139*	-1.699	-2.232^+^	-1.572
	(1.07)	(1.767)	(1.294)	(1.643)
Proportional	-1.424*	-1.420*	0.8	0.823
	(0.585)	(0.585)	(0.57)	(0.574)
Gender (Male = 1)	0.719	0.72	-0.064	-0.097
	(0.486)	(0.486)	(0.576)	(0.583)
Super senior	-2.933*	-2.849*	1.428*	1.380^+^
	(1.21)	(1.221)	(0.71)	(0.715)
20th Term	1.318***	1.320***	0.59	0.578
	(0.301)	(0.302)	(0.418)	(0.418)
betweenness	-0.004	-0.004	-0.129*	-0.131*
	(0.022)	(0.022)	(0.064)	(0.064)
Loyalty:Low Participants		0.139		0.24
		(0.476)		(0.477)
Constant	-1.361**	-1.363**	-1.547*	-1.505*
	(0.527)	(0.528)	(0.647)	(0.653)
Observations	247	247	208	208
Log Likelihood	-140.201	-140.159	-82.308	-82.188
Akaike Inf. Crit.	296.403	298.319	180.617	182.375

Note: +p<0.1

*p<0.05

**p<0.01

***p<0.001

The dataset for the ruling party yields significant results for the rewarding model, whereas the opposition party dataset aligns with the sanction model. In Model 1, we run a regression analysis of party loyalty and its reward, i.e., single nomination, using the sample of ruling party members. In Model 3, we examine the causal relationship between high loyalty and party sanction, measured by cut-off, for opposition party members, regardless of their ideology. We present the results for both models, with and without interaction terms. Notably, incorporating the interaction between loyalty and low participation rates enhances the model fit, as evidenced by the significance levels of the loyalty variables in Model 2 and Model 4.

As anticipated, our primary independent variable (loyalty) exhibits a negative coefficient in both models with different dependent variables, which have contrasting implications. This indicates that loyalty has a distinct impact on party status, regardless of the lawmakers’ ideology. Specifically, if a lawmaker displays less loyalty to their ruling party, their chances of receiving a benefit, such as a single nomination, increase. Conversely, those members who exhibit less loyalty to their party are more likely to be deprived of the opportunity to run for the election when they are in the opposition. In other words, the two outcomes demonstrate that members who are less loyal to their party obtain an advantage from their actions when they belong to the governing party, while they are penalized by their leaders and forfeit reelection opportunities when they are in the opposition. However, we did not find statistically significant values in the cutoff model with the ruling party dataset and single nomination model with the opposition party dataset.

We also discovered a statistically significant correlation between electoral nomination and long-serving lawmakers, or "super seniors." The coefficients in model 1 and model 3 indicate that parties are likely to deprive long-serving members of the opportunity for reelection. Among ruling party members, there is a significant negative relationship between single nomination and having served as a member of the National Assembly several times, which indicates that parties impose strict nomination standards for these lawmakers. Similarly, super seniors are more likely to be excluded from the nomination process when they are in the opposition group. In Korean politics, seniority is generally viewed as having been in power for too long, which the general public does not appreciate. The negative relationship with the chance of reelection suggests that political parties in Korea are strategically involved in the nomination process to increase their odds of winning the election. For the same reason, those who are elected in a district (Proportional equals 1) are also less likely to receive a single nomination when they belong to the ruling party. Lastly, the negative significant effect of betweenness centrality on cut-off among opposition groups suggests that bridging within the party is valued, while distancing from the other party is crucial, given the significance of loyalty in the opposition party.

**Fig 3:**
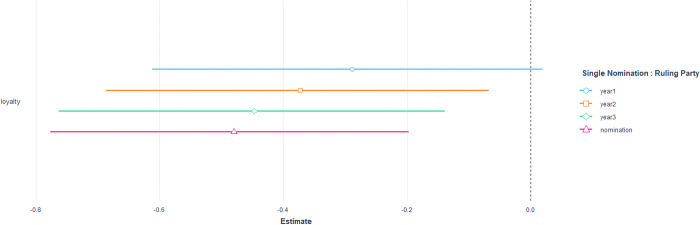
Expected coefficients and 95% of standard error of loyalty from rewarding model with ruling party members. Each line indicates the loyalty extracted from the bill sponsoring data extracted by the year.

To investigate the effect of loyalty duration on the likelihood of nomination, we divided the loyalty data for individual lawmakers into four different time periods. We then fit models 2 and 4 with the corresponding datasets after adjusting the loyalty measure for the different time periods. Fig 3 presents the results for the single nomination model with the ruling party members, which shows statistical significance in the cumulative loyalty variable from the second year onwards. This result supports our earlier regression table findings. The figures also provide insights into the timing of the nomination decision-making process, which occurs in the early stages of the Assembly term and continues until the nomination announcement when the party is in power.

**Fig 4:**
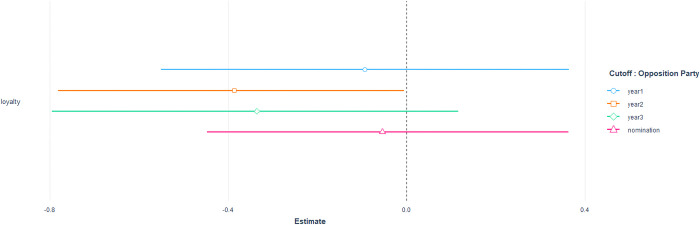
Expected coefficients and 95% of standard error of loyalty from sanction model with the opposition party members. Each line indicates the loyalty extracted from the bill sponsoring data extracted by the year.

Unlike the single nomination model, Fig 4 presents the results of the cut-off models with the opposition party, which show a slightly different aspect than the former. Only the party loyalty data extracted from up to the second year show statistically significant features, and there are no meaningful results in the other datasets. This suggests that the causal relationship between party disloyalty and strong punishment, specifically cut-off from the nomination, occurs for a very short period, primarily in the early days of the National Assembly. Alternatively, we can interpret this as a phenomenon where lawmakers are intentionally manipulating loyalty to avoid punishment or for other reasons.

## Concluding remarks

This paper investigates the relationship between party disloyalty and ideological polarization in the legislative branch, with a focus on the moderating effect of a political party’s status (ruling or opposition) in the National Assembly. The central hypothesis is that ruling parties are more likely to endorse disloyal candidates with issue positions that diverge from their own platform, while opposition parties tend to punish disloyal candidates to demonstrate party unity during the nomination process. To test this hypothesis, we employ data from the 19th and 20th South Korean National Assembly, where party leaders dominate the nomination process for parliamentary elections. To measure the degree of loyalty of lawmakers, we adopt the SDSM Backbone Networks method, as only a fraction of bills in South Korea are subject to roll-call votes, necessitating an alternative measure of ideological polarization in the National Assembly. Using SDSM, we extend existing bipartite network approaches to estimate the structural positions of lawmakers based on a more realistic probability distribution. In so doing, we contribute to the literature on ideological polarization at the elite level, both methodologically and substantively.

The overall findings of this study are generally in line with the hypotheses put forth. The analysis reveals that, within the ruling party, individuals who are loyal to the party are less likely to receive preferential treatment during the nominating process compared to those who are not party loyalists. Additionally, the likelihood of loyalists receiving nominations consistently decreases as their track records progress towards the next election cycle.

On the contrary, within the opposition party, party loyalists are less likely to be excluded from the nomination process. However, this particular result attains statistical significance only when their track records up to the second year following the election are taken into consideration.

In the past, majority-ruling parties have typically won general elections and maintained their majority status. However, the balance of power in the National Assembly has only shifted to the opposition party when they have won the preceding Presidential election, with one exception occurring in the 20th general election when the Saenuri party lost its majority status by only one seat. This was largely due to the success of a third party, the People’s Party, which secured 35 seats and became the most successful centrist political party in Korean history. Therefore, although the Saenuri party’s decision to favor non-loyalists in the election was ultimately unsuccessful, it can be argued that it was a well-reasoned choice given the circumstances.

Overall, these findings are consistent with previous research that highlights the significant role of party loyalty in driving political polarization [46, 47]. In the context of Korean politics, our study indicates that there are clear incentives and experiences for ruling parties to prioritize politicians who can mitigate polarization. Conversely, minor opposition parties tend to support individuals who contribute to the intensification of polarization. To comprehensively comprehend party polarization among political elites, it is crucial to acknowledge the diverse motivations underlying the behavior of party members in candidate selection. These motivations may vary depending on whether the party holds the status of the ruling party or the opposition party.

Some limitations of this study are worth mentioning. First, our data were limited to two terms (the 19^th^ and 20^th^ National Assembly). While we are open to extending our analysis to the previous general elections, our theoretical question is particularly relevant to these two terms because political power structure between the ruling and opposition parties was relatively balanced, as opposed to the previous ones. The power balance was likely to allow the competing political parties to moderate their ideological polarization and bridge gaps [48].

Second, the limited time coverage of this study does not allow for a nuanced comparison between the three-Kims era and the present, which will be sorely missed by those who are well-conversant with the contemporary Korean politics. During the three-Kims era, party loyalty was directed to one person (boss), who virtually monopolized the nomination power. In the post-three-Kims period, political parties are more fragmented and decentralized, and therefore party loyalty is more likely to be oriented to party ideology, which is usually represented by the dominant faction of the party. Disloyal members can still be punished by being excluded from the nomination, but it is not likely to occur if they belong to a major faction or if they entertain a reasonable amount of support from ordinary citizens, who are not party members. Empirical evidence is needed to corroborate this account.

Third, issues regarding campaign finances, which may be a function of party members’ loyalty, are not discussed in the present study. In Korean electoral politics, money is strictly controlled by laws. Unlike the United States, for example, candidates and political parties can neither spend nor receive money freely. Voters cannot donate money freely, either. It does not necessarily mean that Korean politicians are not connected with the rich people and corporations. But promoting interests of the big donors, if any, is an extremely risky act in the polarized era because it is highly likely to be detected and persecuted. The nature of money in politics makes it difficult to gather reliable data, which is a pre-condition for further research.

Future research has to address power dynamics within a political party in a more nuanced manner. Party elites are influential, but they are not omnipotent. Party leaders usually exert influence on nomination process. Up until the dawn of the 21^st^ century, illegal flow of money to party leaders was widespread in nomination process, but it is not the case anymore. The nomination power of party leaders is now checked and balanced by the following: (1) factions within a political party and (2) the usage of public opinion polls in electing party leader.

First, factions exist in the Korean political parties. In the ruling party, a faction comprised of politicians close to the president is likely to be at odds with other factions whose members are not close to the president. As of now, the president is not allowed to explicitly engage him/herself in within-party politics. Nevertheless, political power of the president is huge enough to build a faction in the ruling party. Other factions in the ruling party are interested in fostering presidential candidates for the next election cycle, because reelection of the president is strictly prohibited by the Korean Constitution. The situation is basically the same in the opposition party: multiple factors compete with each other to nominate their preferred politician as the presidential candidate.

Second, political parties have recently relied on public opinions in electing party leaders. Theoretically, it does not make sense for political parties to consider preferences of ordinary citizens in within-party elections, which must be allowed only for party members. However, as political parties eventually have to appeal to ordinary voters to succeed, they come to believe it is helpful to include both party members and ordinary voters in the process of selecting party leadership. How to weight them (e.g., 70% party members and 30% non-members or 50% party members and 50% ordinary citizens) matters. Since party leaders are not able to fully control non-members, nomination process is less likely to be dominated by them as the portion of public opinion polls increases.

One may believe that the external validity of this study is somewhat damaged by the recent changes of electoral laws in South Korea. The electoral reform in 2019 originally intended to increase proportionality by assigning seats of local districts in proportion of the total number of votes gained by political parties. The simplest way in which one can increase proportionality is to replace single-member local districts with proportional seats. Instead of doing this, the 2019 reform allowed to consider the total number of votes secured by political parties in assigning the number of seats. The total number of seats (300) remains the same. The number of single-member local districts (253) and proportional seats (47) do not change at all. The only difference is that the assignment of both (a portion of) seats from local districts and proportional seats becomes a function of the total number of nationwide votes gained by political parties from 2019 on. (Before, only the assignment of proportional seats was subject to the total number of votes.) That said, the 2019 electoral reform was not sufficient to increase proportionality by design. It is true that the reform led to the rise of the satellite offshoots (a.k.a., bloc parties), but as most of their members are absorbed by the major political parties, the reform’s impact on party loyalty seems to be almost non-existent.

## Supporting information

S1 File Description of extracting backbone relationship of politicians with SDSM. (PDF)

S1 Fig Visualizing the construction of the BACKBONE relationship extraction framework. Our diagram showcases the process of constructing the BACKBONE framework, highlighting the stochastic nature of the upper section and comparing stochastic results with observed values in the lower distribution. This visualization effectively demonstrates the framework’s ability to distinguish positive and negative examples in relationship extraction.
(TIF)
