# Supplementary material for: Do political parties always prefer loyalists? Evidence from South Korea
Source: PLoS One. 2023 Nov 2;18(11):e0291336. doi: 10.1371/journal.pone.0291336 (PMC10621924; doi:10.1371/journal.pone.0291336)
Supplement: S1 File — (PDF) [file pone.0291336.s001.pdf]

Supporting Information

Stochastic Degree Sequence Model(SDSM)

The general framework to extract the backbone network using Stochastic Degree sequence Model(SDSM) follows three steps. In the first step, the probability of a certain lawmaker sponsoring a particular bill is predicted with binary outcome models. In the next step, using the output of the prediction, N times of Bernoulli trials are tired if the person signs the bill. The result can be interpreted as an estimated bill sponsor network on each trial and therefore we can obtain the number of estimated joint sponsoring by one mode projection. Lastly, with the cumulated prediction, we make a co-sponsorship distribution for each pair of lawmakers and compare the observed number of cosponsor and null distribution. If the observation is located outside of its confidence interval, the relationship is defined. In backbone network, there are two different types of edges either positive or negative. In some cases, no significant relations are defined from the model and thus that some of the pairs are not linked together which means the observed number of co-sponsorships could occur randomly.

S1 Fig. Visualizing the construction of the BACKBONE Relationship Extraction Framework. Our diagram showcases the process of constructing the BACKBONE framework, highlighting the stochastic nature of the upper section and comparing stochastic results with observed values in the lower distribution. This visualization effectively demonstrates the framework's ability to distinguish positive and negative examples in relationship extraction.

Let B be an observed bipartite network where its row represents the agents and the column represents the artifacts. If Agent i signs artifact k , $B_{ik} = 1$ or zero otherwise. $P = B * B^T$ is the one-mode projection of the bipartite and its element (P_{ij}) shows the number of joint cosponsors from the total number of artifacts. We first fit a binary outcome model predicting a certain lawmaker signing a bill (B_{ik}) as a function of agents' (Assembly member) and artifacts' (Bill) degrees. We then find the estimated agent-to-artifact linkage probabilities (p_{ik}) using the fitted coefficients. Lastly, we generate a

random bipartite network (B*), which is the outcome of one Bernoulli trial using the estimated probability, and we perform one-mode projection (P*) to obtain an estimated co-sponsor network. If we repeat the process N times, we can obtain the conditional null distribution of the number of co-sponsors upon every pair of lawmakers. This distribution draws the expected number of co-sponsors under the assumption of the null hypothesis that the two lawmakers propose a bill in a random manner conditioning the degree of agent and artifacts. We then compare the observed number of joint sponsorships with the empirically derived null distribution, with decisions about the statistical significance of an edge made using a two-side t-test with a significance level of 0.05. The relations are inferred if the observation is not located within the confidence interval in the null distribution generated by SDSM. A positive relation exists when the observed number of co-sponsors is significantly larger than the expected under the null hypothesis, and in the case of a negative relationship, vice versa. Here, depending on where the observations correspond in the conditional distribution, the relationship between the two representatives can be identified as positive, negative or moderate. In some cases, no significant relations are defined from the model and thus some of the pairs are not linked together, which means the observed number of co-sponsorships could have occurred randomly.

We adopt the Bipartite Configuration Model (BiCM) to fit a binary outcome model to obtain the predicted probability. BiCM is a reference model of SDSM to generate a statistical null distribution for binary bipartite networks. It offers an unbiased method for analyzing node similarities and obtaining statistically validated one-mode projection[1, 2].

Appendix

1. Saracco F, Di Clemente R, Gabrielli A, Squartini T. Randomizing bipartite networks: the case of the World Trade Web. Sci Rep. 2015;5:10595.
2. Domagalski R, Neal Z, Sagan B. backbone: An r package for backbone extraction of weighted graphs. arXiv preprint arXiv:191212779. 2019.
