# Supplementary figures and images for: Do political parties always prefer loyalists? Evidence from South Korea
Source: PLoS One. 2023 Nov 2;18(11):e0291336. doi: 10.1371/journal.pone.0291336 (PMC10621924; doi:10.1371/journal.pone.0291336)

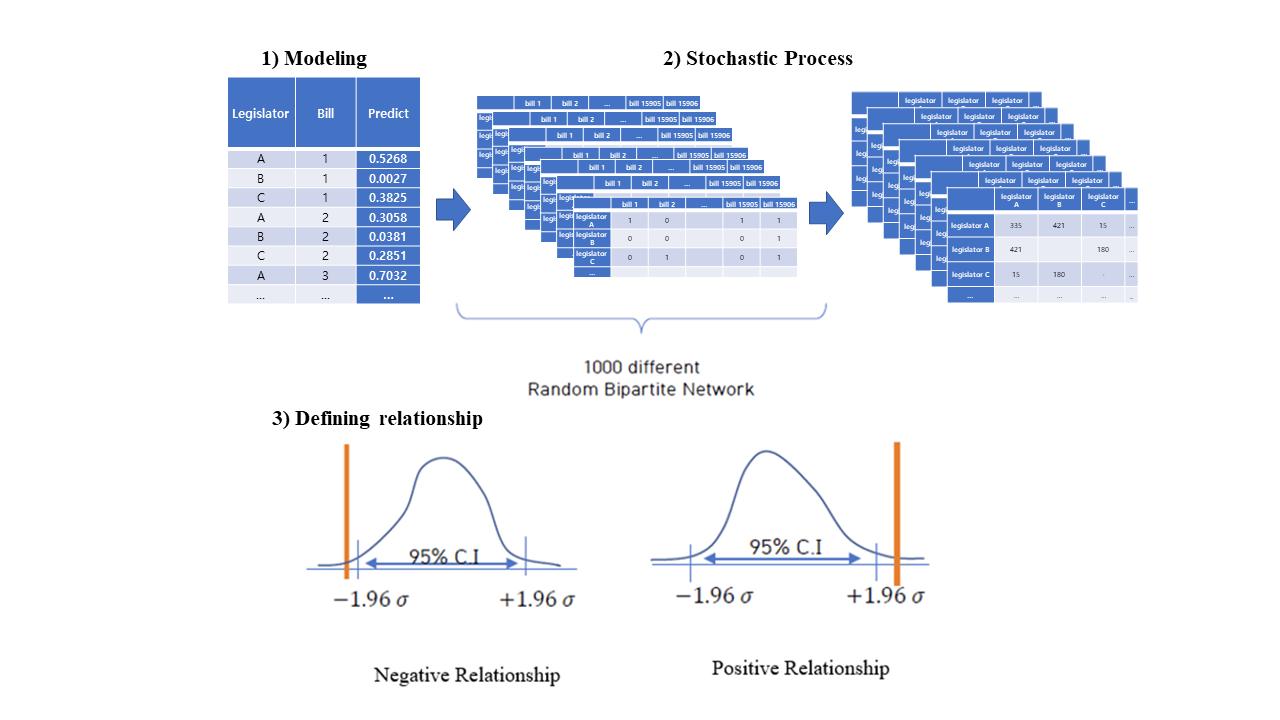

Supplement: S1 Fig — Our diagram showcases the process of constructing the BACKBONE framework, highlighting the stochastic nature of the upper section and comparing stochastic results with observed values in the lower distribution. This visualization effectively demonstrates the framework’s ability to distinguish positive and negative examples in relationship extraction. (TIF) [file pone.0291336.s002.tif]
